# Which Current and Novel Diagnostic Avenues for Bacterial Respiratory Diseases?

**DOI:** 10.3389/fmicb.2020.616971

**Published:** 2020-12-10

**Authors:** Héloïse Rytter, Anne Jamet, Mathieu Coureuil, Alain Charbit, Elodie Ramond

**Affiliations:** ^1^Université de Paris, Paris, France; ^2^INSERM U1151, Institut Necker-Enfants Malades. Team 7, Pathogenesis of Systemic Infections, Paris, France; ^3^CNRS UMR 8253, Paris, France; ^4^Department of Clinical Microbiology, Necker Enfants-Malades Hospital, AP-HP, Centre Université de Paris, Paris, France

**Keywords:** lung disease (diagnosis), respiratory tract infection, diagnostic test, artificial intelligence, rapid test

## Abstract

Bacterial acute pneumonia is responsible for an extremely large burden of death worldwide and diagnosis is paramount in the management of patients. While multidrug-resistant bacteria is one of the biggest health threats in the coming decades, clinicians urgently need access to novel diagnostic technologies. In this review, we will first present the already existing and largely used techniques that allow identifying pathogen-associated pneumonia. Then, we will discuss the latest and most promising technological advances that are based on connected technologies (artificial intelligence-based and Omics-based) or rapid tests, to improve the management of lung infections caused by pathogenic bacteria. We also aim to highlight the mutual benefits of fundamental and clinical studies for a better understanding of lung infections and their more efficient diagnostic management.

## Introduction

**FIGURE 1:**
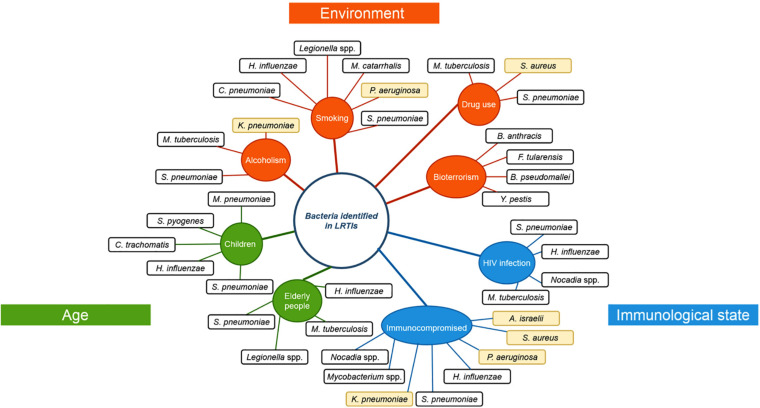
Risk factors and their associated respiratory pathogens. Risk factors that were observed to favor specific respiratory pathogen infections. Risk factors are classified according to their type of risk: age (green), environmental influence (orange) or immunological state (blue). Opportunistic pathogens are highlighted in yellow. *H. influenza: Haemophilus influenza, C. pneumonia: Chlamydophila pneumoniae, M. catarrhalis: Moraxella catarrhalis, S. pneumoniae: Streptococcus pneumoniae, P. aeruginosa: Pseudomonas aeruginosa, K. pneumoniae: Klebsiella pneumoniae, M. tuberculosis: Mycobacterium tuberculosis, M. pneumoniae: Mycoplasma pneumoniae, S. pyogenes: Streptococcus pyogenes, C. trachomatis: Chlamydia trachomatis, S. aureus: Staphylococcus aureus, A. israelii: Actinomyces israelii, Y. pestis: Yersinia pestis, B. pseudomallei: Burkholderia pseudomallei, F. tularensis: Francisella tularensis, B. anthracis: Bacillus anthracis*.

The human respiratory tract is divided in two spatial environments: the upper respiratory tract (URT), including tonsils, nasopharynx, oral cavity, oropharynx, and larynx; and the lower respiratory tract (LRT), including trachea, bronchi, and lungs. The composition of the LRT microbiota, which was considered until recently as a sterile environment, has been revealed by the development of advanced genomic sequencing techniques (Charlson et al., 2011) and showed notably the presence of Proteobacteria, Firmicutes, and Bacteroidetes (Huang et al., 2013; Yu et al., 2016). This microbial community is the result of equilibrium between acquisition of bacteria through inhalation and elimination (lung clearance) involving mucociliary blanket movements and immunity. It is also shaped by many host factors (such as the age, immunological status, genetic background, physiological parameters, nutrient availability, etc., described in Figure 1) and impacted by antibiotic treatments, anti-inflammatory compounds or underlying diseases (Venkataraman et al., 2015; Rali et al., 2016). In addition, socio-demographic and socio-cultural aspects contribute to the acquisition of new micro-organisms (Savitha et al., 2007; Miyashita et al., 2018).

**FIGURE 2:**
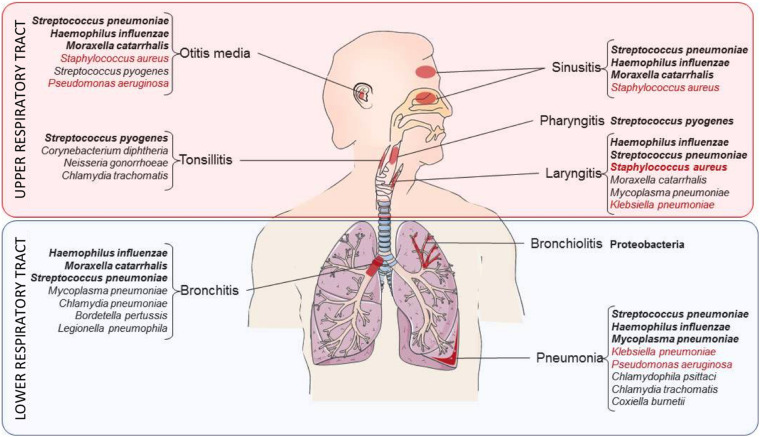
Bacteria responsible for the most common respiratory diseases. Summary of the bacteria responsible for respiratory infections in the URT (red zone) and the LRT (blue zone). Bacteria are grouped according to the respiratory niche they preferentially infect. Pathogens indicated in bold correspond to the main bacteria encountered in airways while the ones in red correspond to opportunistic pathogens.

LRT can become the reservoir of bacterial pathogens [e.g., *Mycobacterium tuberculosis* (MTB), *Streptococcus pneumoniae*, *Legionella pneumophila, Staphylococcus aureus, Haemophilus influenzae* and many others, shown in Figure 2], that can lead to dramatic clinical outcomes (Savitha et al., 2007; Rali et al., 2016). In 2016, LRTIs were the major cause of death considering all age groups (including patients that are less than 5 years-old and more than 70 years-old). Malnutrition, air pollution as well as antibiotics overuse were identified as aggravating factors (GBD 2016 Lower Respiratory Infections Collaborators, 2018). Furthermore, many bacterial pathogens involved in LRTIs are multi-resistant to antibiotic treatments and are considered as priority agents for research and development by the World Health Organization [WHO report on antibacterial agents in clinical development (World Health Organization, 2019)]. Among them, MTB, the causative agent of tuberculosis, represents the number one global infectious disease killer today, causing 1.8 million deaths per year (Furin et al., 2019).

To circumvent the use of broad-spectrum empirical antimicrobial therapy, that is clearly identified as a cause of multidrug resistant bacteria emergence and spreading (Leekha et al., 2011), the development of novel fast and easy-to-use diagnostic technics is required (Caliendo et al., 2013). Major advances have been done in the past years in the field of diagnosis to identify lung bacterial pathogens, in terms of accuracy and speed. In this review, we have chosen to first recall the different methods of sampling, how clinicians can quickly determine the bacterial or viral origin of pneumonia and the actual tests that are routinely used for pathogen detection, Then, we will focus on recent technological approaches and their potential applications in the field of diagnosis of bacterial lung infections. This includes technologies based on image analysis and rapid diagnostic tests.

## Current Methods and Tools for Pathogen Identification

### Specimen Collection for Pneumonia Diagnostic and Traditional Screening Methods

Diagnosis is defined as the identification of the nature of an illness by examination of its symptoms and signs. In the case of URT infections, such as otitis media and tonsillitis, a clinical examination is often sufficient to diagnose the disease and can lead to simple local microbiological sampling. In contrast, it is more complicated to detect and analyze LRTIs such as bacterial-related pneumonia based on etiologic examination (Dasaraju and Liu, 1996). This raises the question of sample collection for bacterial identification and appropriate therapeutic treatment administration. Specimen collection includes in first place non-invasive (and generally non-sterile) methods that consist in sputum collection in case of pus producing pneumonia. However, this technique can be more difficult to apply in children as they have difficulties to expectorate. This can be counteracted by sputum induction through inhalation of nebulized sterile saline solution (Lagerström et al., 2004; Grant et al., 2012). Urine collection can also be performed and is easier to ask for, especially among children patients. It allows identifying specific bacterial antigen for *S. pneumoniae* or *L. pneumophila*. On the other hand, invasive techniques propose efficient and mostly sterile alternatives for pathogen identification. In terms of sterile techniques, blood sampling, thoracentesis (in case of pleural effusion, a very common symptom during bacterial pneumonia), trans-thoracic needle aspiration (TNA) and bronchoalveolar lavages (BAL, performed with fiber-optic bronchoscope) are mostly used. Then, specimen culture or polymerase chain reactions (PCRs) can be performed (Menezes-Martins et al., 2005; Loens et al., 2009). Blood sampling can also serve for evaluation of the inflammatory response (hemogram, C-reactive protein, etc…) and is proposed in first intention, especially in children population (Loens et al., 2009). Protected specimen brush (PSB) consists in a thin collection brush protected by a sheath. When the brush reaches the desired area, it is extended to collect lung secretions and cells. Since more then 40 years, it is considered as a reference test for diagnosis in pneumonia by limiting bacterial contamination from the URT (Wimberley et al., 1979; Mertens et al., 1998).

Culture-based diagnostic tests remain the gold standard for organism identification. Cultures are prepared from lung secretions if sample collection is of good quality (Mandell et al., 2007). However, the use of culture technics for pathogen identification can be hampered if samples are contaminated with bacteria from URT (Giuliano et al., 2019) and results are obtained usually in a minimum of 48 h, a delay that could greatly compromise patient outcome. Notably, the main advantage of culture is the possibility to determine antibiotic susceptibility to a wide range of molecules allowing optimal antimicrobial therapy administration.

### How to Differentiate Bacterial Associated-Pneumonia From Virus-Associated Pneumonia

Distinguishing between bacterial pneumonia and viral pneumonia is of great importance, especially to avoid useless antibiotic treatment. Diagnosis can be difficult to make for general practitioners with limited technical resources. Diagnosis can be guided by patient specificities such as severity of disease or patient’s comorbidities and by local epidemiology. Graffelman et al. (2004) proposed a scoring system based on very simple criteria that could help managing patients. They observed a significant association of headache, fever and painful cervical lymph nodes with bacterial LRT infection, in contrast to the association with rhinitis or diarrhea that are more suggestive of viral LRT infection.

Several tests have been set up at the clinical level to confirm the origin of LRTI. C-reactive protein (CRP) is a blood diagnostic marker more often increased in bacteria-infected patients than in virus-infected ones (6 times higher) (Noviello and Huang, 2019; Thomas et al., 2020). CRP is synthesized by hepatocytes in response to acute tissue inflammation. Its production, that is specifically stimulated by interleukins IL-6 and IL-1, may play a role in pathogens opsonization (García Vázquez et al., 2003). It was defined that CRP level above 100 mg/L is suggestive of acute bacterial infection and requires antibiotic treatment. Similarly, procalcitonine (PCT) is also used as a biomarker of acute bacteria-associated pneumonia. Interestingly, PCT dosage can be obtained in less than 1 h. It is synthesized from liver cells or peripheral blood mononuclear cells, in response to tumor necrosis factor (TNF) or IL-6 production (Creamer et al., 2019). Several studies confirmed PCT testing to play key role in lowering mortality, antibiotic consumption and their associated side effects. More specifically, the BioMérieux VIDAS^®^ B.R.A.H.M.S PCT^TM^ test has been developed and was approved by the Food and Drug Administration (FDA, Noviello and Huang, 2019). It allows determining serum or plasma PCT concentration in 20 min. Based on a decision algorithm that follows PCT concentration, patients who have PCT concentration greater than 0.25 ng/mL should be treated with antibiotics (from https://www.biomerieux.fr/diagnostic-clinique/vidasr-brahms-pct).

### Current Rapid Diagnostic Tests

Rapid Diagnostic Tests (RDTs) must be fast, simple to perform and sensitive. They can be performed at the point-of-care and provide a reliable diagnosis within a short period of time. Hence, RDTs are adapted for use in low-resource settings to provide quick answers to urgent clinicians’ needs. Several RDTs for respiratory infections are already available since many years. For example, the urine test for simultaneous detection of *S. pneumoniae* and *L. pneumophila* antigens (ImmuView^®^) is routinely used in cases of suspected pneumonia since more than 10 years (Kazandjian et al., 1997; Gutiérrez et al., 2003). This test can be used on children over 8 years of age and shows higher sensitivity (between 85 and 89%) than other proposed commercial tests (between 72 and 77%)^1^. New RDTs are now emerging and can be divided into two main categories: (i) syndromic tests, to determine the causative agent of the disease; and (ii) specific tests, to confirm the presence of a suspected pathogen. In this section, we will present a non-exhaustive list of diagnostic tools that are either routinely used in health care facilities (and potentially recommended by the WHO) or promising technics that were recently developed (list in Table 1).

**TABLE 1:** List of the rapid diagnostic tests (syndromic and specific tests) currently available to identify LRT pathogens.

**Diagnostic test names**	**Manufacturer or study ref.**
**Syndromic tests (Multiplex PCR)**	
QIAstat-Dx^®^ respiratory panel	Qiagen
BioFire^®^ FilmArray^®^ pneumonia panel plus	BioMérieux
Unyvero LRT panel	Curetis
RespiFinder^®^ SMART 22 FAST	PathoFinder
DendrisChips^®^	Dendris
**Specific tests**	
- ***Mycoplasma pneumoniae***	
LAMP (loop-mediated isothermal amplification) assay	Saito et al., 2005
Ribolest mycoplasma^®^	Asahi Kasei Pharma
Silver amplification immunochromatography (SAI) system	Namkoong et al., 2018
**- *Mycobacterium tuberculosis***	
Xpert MTB/RIF ultra^®^	Cepheid
Loopamp^TM^ MTBC detection kit	HUMAN
FluoroType^®^MTB kit	Bruker
GenoType MTBDRsI^®^ VER 1.0 *1*VER 2.0	Bruker
GenoType MTBDRplus^®^	Bruker
CRISPR-MTB test	Ai et al., 2019
**- *Non-tuberculous mycobacterium***	
Ezplex^®^ MTBC/NTM real-time PCR kit	SML Genetree

#### Syndromic Tests

The syndromic approach is based on the simultaneous search for the most common microorganisms suspected in an infectious disease. Syndromic tests (STs) allow identification of the pathogen in few hours. In general, STs allow the detection of the most common pathogens, including viruses (e.g., orthomyxoviruses, coronaviruses, paramyxovirus, adenovirus, rhinovirus…) and bacteria (e.g., *Mycoplasma pneumoniae, L. pneumophila, Chlamydophila pneumoniae, H. influenzae, Klebsiella pneumoniae. P. aeruginosa, S. aureus, S. pneumoniae*…). The distinction between viruses and bacteria enables an adapted treatment and a reduction in the use of antibiotics.

Many multiplex PCR panels already exist and are available on the market [such as QIAstat-Dx^®^ Respiratory Panel, BioFire^®^ FilmArray^®^ Pneumonia Panel plus (Parčina et al., 2020), Unyvero Lower Respiratory Tract (LRT) Panel (Collins et al., 2020) or RespiFinder^®^ SMART 22 FAST (Hattoufi et al., 2020)]. These technologies can be adapted easily to new targets, although they have variable sensitivity. We will describe briefly only two of these technologies: BioFire FilmArray and DendriChips.

The FilmArray technology is an example of syndromic diagnostic test that is now broadly used in hospitals. It consists of automated multiplex PCRs. More specifically, BioFire^®^ FilmArray^®^ Pneumonia Panel plus system, from BioMérieux (FDA approved in 2018), extracts and purifies all nucleic acids from the unprocessed respiratory sample (BAL, expectorations and endotracheal aspirates) and performs nested multiplex PCR. Dedicated software automatically analyzes endpoint melting curve data and reports whether each pathogen is detected in the sample or not. This technic can identify in a semi-quantitative manner 18 pneumonia-associated bacteria as well as determining 7 resistance markers (e.g., methicillin ad carbapenem resistance genes). Results are obtained in 1 h thus saving days of unnecessary antibiotics treatment, with levels of sensitivity and specificity reaching ≥ 96%^2^.

Pathogen identification is also possible with the DendrisChips^®^ diagnostics tool. This technology proposes a 16S rDNA-based (ribosomal DNA) detection of respiratory pathogens through PCR (Senescau et al., 2018). Using dendrimer-activated glass surface (called dendrislides) allows reaching a 2-fold higher sensitivity (Le Berre et al., 2003) by immobilizing more strongly the probes on the chips. This multiplex technology allows the detection of 11 bacteria causing respiratory tract infections. A diagnostic result is delivered in about 4 h as a predictive value of presence/absence of pathogens, using a decision algorithm based on machine learning methods.

#### Specific Tests

Specific tests can identify single bacteria commonly found in respiratory infections. A new test, named PneumoResp, targets *S. pneumoniae* cell wall polysaccharide and can be directly performed on respiratory samples. A study conducted on 196 children showed that, by comparison to culture and PCR assays, the PneumoResp test showed a sensitivity and negative predictive value of more than 98% on patients’ secretions. By comparison to classical criteria of *S. pneumoniae* pneumonia (combining typical symptoms, X-ray image and culture ≥10^7^ CFU/mL in sputum or nasopharyngeal secretions), the sensitivity and negative predictive value of PneumoResp test on specimens was higher (Haddar et al., 2020). It allows proposing an antimicrobial treatment targeting *S. pneumoniae* at day 0.

#### Specific Tests for *Mycoplasma pneumoniae*

A rapid diagnosis is particularly interesting when dealing with fastidious and slow-growing bacteria. For example, *M. pneumoniae* bacterium is rarely cultured in clinical microbiology laboratories, as it requires weeks of growth resulting in delayed diagnosis and increasing the risk to develop a severe pneumonia (She et al., 2010; Postma et al., 2015). Instead, nucleic amplification-based approaches including loop-induced isothermal amplification (LAMP, targets stem–loop DNA structures) (Saito et al., 2005) or PCR (Di Marco, 2014) are usually addressed at hospital with results within 3–4 h. To further improve the speed of diagnosis (within 15 min), colloidal gold-based immuno-chromatographic antigen assays, on nasopharyngeal swab or sputum samples, targeting the membrane protein P1 (Li et al., 2015) or L7/L12 ribosomal protein (Ribotest Mycoplasma^®^, Asahi Kasei Pharma, Tokyo, Japan) (Miyashita et al., 2016) are suggested, however these technics show low sensitivity (around 70%) and are highly recommended when patients show several clinical symptoms. More recently, silver amplification coupled with immuno-chromatography has been proposed. Silver amplification assay combines the formation of sandwich immune system with gold nanoparticles (GNP) and silver enhancement. The silver enhancement is based on the reduction of silver ions on the surface of GNP, and provides a significant increase of initial GNP-caused coloration (Namkoong et al., 2018). Test sensitivity and specificity are higher as they reach, respectively, 90 and 100% compared to PCR approaches and thus may be helpful for directly initiating appropriate antibiotic treatment, since this bacterium is naturally resistant to β-lactams (the most common probabilistic treatment).

#### Specific Tests for *M. tuberculosis* (MTB)

Every year, 10 million people contract tuberculosis [TB, WHO report (World Health Organization, 2019)]. In developing countries, about 7% of all deaths are attributed to TB (Zaman, 2010). In low- and middle-income countries, where access to health care is difficult, cheap and easy-to-use RDTs are of great value. Among the specific tests currently available to identify MTB, and that do not imply culture technics or targeted PCR, we will highlight the Xpert MTB/RIF Ultra, TB-LAMP, and GenoType MTBDR*plus* (Eddabra and Ait Benhassou, 2018) that are commonly used and recommended by the WHO. We will briefly recall their characteristics and respective advantages.

Xpert MTB/RIF Ultra is a cartridge-based detection of MTB and its associated rifampicin (RIF) resistance (Cepheid, Sunnyvale, CA, United States). It consists in an automated nested real-time amplification in cartridge using the GeneXpert platform, in 2 h. Fluorescent probes target the *rpoB* gene (RNA polymerase β-subunit) that is responsible for RIF resistance (Arend and van Soolingen, 2018). Since 2011, the WHO recommends its use as an initial diagnostic test if tuberculosis is suspected (World Health Organization, 2013). While this technology gives high sensitivity and specificity (close to 100% compared to standard PCR) to detect MTB in adults, MTB detection is much more challenging in children due to insufficient sample quantity and the scarcity of bacteria in specimen (López Ávalos and Prado Montes de Oca, 2012). Indeed, tests provide sensitivity ranging from 40 to 100% and specificity ranging from 93 to 100% regarding the different studies that have been carried out (World Health Organization, 2013).

TB-LAMP test, using the commercial molecular assay Loopamp^TM^ MTBC Detection Kit, is officially recognized by the WHO Guideline Development Group (GDG) to detect MTB in LRTIs (World Health Organization, 2016). TB-LAMP requires few infrastructures and gives a result regarding MTB detection within 2 h (Iwamoto et al., 2003). However, it is unable to detect drug resistant patterns. The sensitivity of TB-LAMP is greater than smear microscopy that is still the only laboratory diagnostic test for pulmonary TB in low- and middle-income countries (Pham et al., 2018).

FluoroType^®^ MTB kit (Bruker, Germany) is based on DNA amplification through the use single-stranded amplicons labeled with fluorescent probes. This technics show high sensitivity and specificity compared to reference culture methods (respectively 94 and 100%) in respiratory samples (Hofmann-Thiel et al., 2016).

One critical aspect to address is the quick detection of MTB with multidrug-resistance properties. The WHO has addressed recommendations for the use of molecular genetic tests called “second-line line probe assays” (SL-LPA) to identify these types of strains. More specifically, the WHO strongly suggests using the GenoType MTBDR*sl* VER 1.0 or VER 2.0 (Bruker, Germany) as they both detect MTB mutations associated with drug resistance. These tests are based on a DNA strip technology (designed by Hain Lifescience in the 2000s) that consists in multiplex DNA amplification with biotinylated primers, followed by DNA denaturation that yields single-stranded amplicons. After binding to their complementary strand on the strip, amplicons are revealed through alkaline phosphatase treatment that will generate a visible dye. GenoType MTBDR*sl* tests target mutations in: (i) *gyrA* (VER 1.0 and VER 2.0) and *gyrB* (VER 2.0) (encoding *DNA gyrase A* and *B*, involved in fluoroquinolone resistance), (ii) *rrs* (VER 1.0 and 2.0) and *eis* promoters (VER 2.0) (encoding a 16S ribosomal RNA and an aminoglycoside acetyltransferase, involved in amikacine/kanamycin resistance), (iii) the *embB* gene (VER 1.0) (encoding an arabinosyltransferase involved in ethambutol resistance). In addition, the GenoType MTBDR*plus* (Bruker, Germany) allows the detection of MTB carrying resistance to both rifampicin (RIF) and isoniazid (INH) antibiotics by targeting significant mutations in *rpoB* gene (coding for the β-subunit of the RNA polymerase), the *katG* and *inhA* genes [encoding the catalase and an NADH enoyl ACP reductase, providing each resistance to high and low isoniazid concentrations (Nathavitharana et al., 2016)].

Of note, a new RDT, based on CRISPR technology, was also recently developed and tested on a cohort of 179 patients: the CRISPR-MTB test (Ai et al., 2019). This test combines a recombinase polymerase amplification step and a following Cas12a detection step for target detection of S6110, a MTB-specific insertion sequence presents 6–10 times per genome. The CRISPR-MTB test showed a greater sensitivity over both culture and Xpert detection method, and a specificity of 98%. Hence, this new MTB test offers great potential as a new diagnostic technique for pulmonary TB.

Non-tuberculous mycobacteria (NTM, 140 species reported), that are not related to MTB but are also responsible for pneumonia such as *M. avium* or *M. kansassi*, can also be tested through Ezplex^®^ MTBC/NTM Real-time PCR kit. In 2 h, this kit can test samples for the presence of 100 different NTM species and shows high sensitivity and specificity (96–100%) compared to classical PCR or culture tests (Lee et al., 2018).

In conclusion, RDTs allow a rapid and efficient diagnosis of bacterial lung infections. Furthermore, some of them also provide information on the antibiotic resistance pattern, and may therefore potentially limit multi-resistant strains emergence.

New RDTs are constantly developing either from novel technologies or from the improvement of classical RDTs approaches. For example, the Volatile Organic Compound (VOC) Profiling technology appeared in the 1990s and has been developing ever since for diagnosis. The use of the “electronic nose” allows to detect VOCs directly via a portable gas chromatography device coupled with ion mobility spectrometry (GC-IMS) and recently allowed bacterial RTI diagnosis from exhaled breath of hospitalized patients (Lewis et al., 2017). This promising technology is a simple non-invasive, safe and fast method when it is carried out by a specialist, but its sensitivity and specificity still need to be optimized (Schnabel et al., 2015; van der Schee et al., 2015). Of note, it also requires data pre-processing (machine learning) with breathomics results (Smolinska et al., 2014) which can explain the current relative low reliability of this method. Altogether, these parameters suggest that this methods is difficult to implement in health care facilities, due to the lack of specialized employees and the time consuming analysis (van Oort et al., 2017).

## Innovation in Diagnostics for Bacterial Pulmonary Infections Management

### Technologies Based on Image Analysis

Chest X-rays (CXRs) and computed tomography (CT) are crucial for physician to diagnose lung infection and are essential to evaluate pneumonia evolution as well as complications. However, their interpretation may prove difficult as CXRs and CT can neither determine the nature of the infectious agents (viral or bacterial), nor their specificities (susceptibility profile, pathogenicity) and radiographic readings can be influenced by the immunological status of the patient (Franquet and Chung, 2019). Hence, diagnostic methods for rapid and efficient management of lung infections through medical imaging are necessary.

Artificial intelligence (AI), which refers to systems that imitate human thought and actions, is now broadly used to help interpret CXRs for the diagnosis of respiratory infections. X-ray imaging is the most common and available diagnostic technique used in the world, however specialist workers are not necessarily trained in advanced analysis and this led to the development of AI-aided strategies that support clinicians, with the advantage of limited cost (Hashmi et al., 2020). Machine learning (ML) and deep learning (DL) are part of AI and have garnered a lot of attention over the past 2 years. ML is defined as the application of statistical methods to define algorithms. The machine can be constantly “fed” by humans for greater efficiency, with data on one side and solutions on the other side, with the aim of being able to classify new examples (Mekov et al., 2020). DL is a branch of the learning machine, defined by a system that learns via neural networks (networks of algorithms) without human guidance. To perform these analyses, several convolutional neural networks (CNN or ConvNet) have been designed. They consist in multi-layer neural networks that recognize visual patterns from pixel images (Shin et al., 2016). Among the different tools recently developed, the existing CNNs CXNet-m1 (Xu et al., 2019), CheXNeXt (Rajpurkar et al., 2018), VGG16 and VGG19 (Toǧaçar et al., 2020), AlexNet (Rahman et al., 2020; Rajaraman and Antani, 2020; Toǧaçar et al., 2020), ResNet18 (Rahman et al., 2020; Rajaraman and Antani, 2020), DenseNet201 (Rahman et al., 2020), SqueezeNet (Rahman et al., 2020), VGGNet (Rajaraman and Antani, 2020), GoogLeNet (Saraiva et al., 2019), Lastly, Hashmi and collaborators proposed the most accurate and precise model regarding previous developed programs (Hashmi et al., 2020) using ResNet18, Xception, InceptionV3, DenseNet121 and MobileNetV3 CNN algorithms. They could develop a robust model for bacterial pneumonia detection with the help of hospital-scale CXR and CT databases provided respectively from Wang et al. (2017) (named ChestX-ray 14) and Kermany et al. (2018). All the CNNs enumerated here showed high performance metrics in terms of accuracy. However, their respective performances are difficult to estimate as their respective scores (in terms of accuracy, sensitivity, specificity, AUC, etc… are tested against parameters intrinsic to each study, involving CNNs or humans. In a recent paper that compared CXNet-m1 and VGG16 (which are among the first proposed CNNs in the literature), authors showed that they reach, respectively, 96.3 and 96.2% of success in pneumonia identification, without distinction between bacterial or viral origin (Rahman et al., 2020). Of note, differentiating the type of pneumonia results in loss of performance. Indeed, the authors identified DenseNet201 as being the most accurate network to detect pneumonia (reaching 98% of accuracy) but this accuracy dropped down to 93% when it had to differentiate between bacterial and viral origin.

Learning techniques have also been optimized for pulmonary-thoracic segmentations and improvement of pneumonia diagnosis in pediatric medicine through chest radiographs (Longjiang et al., 2019). Indeed, analysis of lung shape changes or size measurement can provide direct diagnosis or clues for serious diseases such as cardiomegaly and pneumothorax. However, the diverse lung shapes of children make lung segmentation in pediatric CXRs considerably more challenging than that of adults. To circumvent this difficulty, an algorithm has been developed, based on the pulmonary-thoracic ratio, that aims at defining accurate lung segmentation to discriminate between bacterial and viral pneumonia, hence helping in the rapid implementation of an adequate treatment (Longjiang et al., 2019).

Lung ultrasound is an alternative technology to X-ray. A recent study demonstrated that it was possible to train an artificial neural network to detect evidence of pneumonia infiltrates through lung ultrasound pictures, collected from young hospitalized children with a diagnosis of pneumonia (Correa et al., 2018). This method achieved a high level of success to detect pattern associated with pneumonia infiltrates (sensitivity of 90.9%, specificity of 100%). This non-ionizing technology might be applied to more portable and less expensive ultrasound devices and be brought to remote, rural areas, where diagnosing pneumonia is frequently a challenge.

Overall, AI-based image analysis represents a promising technology in the automatic clinical diagnosis of bacterial lung infections, as it can assist in clinical decision-making by quickly transforming complex data into more actionable information. This type of diagnostic may act as a “second opinion” to help clinicians and radiologists to evaluate pneumonia severity correlated with a specific pathogen.

### Omics Approaches

The development of “Omics” technologies has considerably improved our knowledge on bacterial genetic diversity and on fundamental mechanisms of bacterial pathogenicity (Jean Beltran et al., 2017). Omics refers to the set of technologies that allows the global understanding of complex and dynamic biological systems through analysis of genes (genomic), RNA (transcriptomic), proteins (proteomic) or metabolites (metabolomic). Now, these high-throughput technologies are also used to develop novel diagnostic approaches. Here, we will focus on the uses and potential of metabolomics in the diagnosis of RTIs.

#### Metabolomics

Metabolomics consists in the analysis of the whole metabolome i.e., the sum of all low molecular weight molecules (<1,500 Daltons) inside a cell, tissue or organism in a given set of physiological, pathological or environmental conditions. Metabolomics can be used as prognostic tool via the discovery of biomarkers. For instance, in patients with severe bronchiolitis, which requires positive pressure ventilation (PPV), serum metabolomic profiles have been shown to differ depending on severity (Stewart et al., 2019). Hence, this approach should allow determining more rapidly the risks of evolution toward severe bronchiolitis.

Interestingly, urine sampling for metabolomic scanning can be also of great help for bacteria identification. A recent metabolomic study of urine (Del Borrello et al., 2020) showed that it was possible to distinguish bacterial from viral causes of pediatric community-acquired pneumonia by searching for about 20 metabolites, such as androstenedione (a testosterone precursor) and pregnanediol (a derivative of progesterone), which are significantly increased in the case of pneumococcal infection.

Numerous studies focused on the use of metabolomics in the search for new biomarkers of pulmonary TB (Lau et al., 2015; Preez et al., 2019). One of the complications of TB, especially in HIV (Human Immunodeficiency Virus) patients, is called TB-associated immune reconstitution inflammatory syndrome (TB-IRIS). This syndrome results in the paradoxical appearance or aggravation of TB-related clinical symptoms (fever, adenopathy, pleurisy, pulmonary infiltrate) consecutive to treatment initiation after an initial improvement. A pilot untargeted metabolomic study showed that it was possible to distinguish between HIV-TB patients with and without TB-IRIS (Silva et al., 2019) by comparing their arachidonic acid, linoleic acid and glycerophospholipid metabolism in plasma samples with liquid-chromatography mass spectrometry.

In conclusion, metabolomic approaches of biological samples (such as urine, bronchoalveolar lavage fluids, plasma…) should lead to the identification of new biomarkers that could be used in future rapid tests, allowing appropriate therapeutic management.

#### Metagenomics

Next Generation Sequencing (NGS) is a biotechnological revolution, allowing the sequencing of large quantities of DNA or RNA in record time. The use of NGS in the hospital environment is promising and many studies adopt this approach to identify pathogens in various respiratory infections. Metagenomic sequencing (mNGS) allows the identification of organisms without having a target in mind and potentially some of their functional characteristics by directly sequencing nucleic acids from respiratory samples (Lecuit and Eloit, 2015). It can be assessed from BAL, transtracheal aspiration or pleural effusion and it requires avoiding contamination (Takeuchi et al., 2019). Metagenomic sequencing includes on one hand targeted metagenomics where there is an initial step of PCR (usually targeting 16S rRNA gene) before sequencing. On the other hand, shotgun metagenomics relies on the sequencing of DNA of both human host and microorganisms without amplification step.

Illumina technology, the most employed sequencing technology in the world, allows obtaining sequences in 2–3 days. In contrast, the nanopore-based MinION sequencing is faster allowing identification of bacterial species and antibiotic susceptibility profile within a day (Charalampous et al., 2019).

One of the advantages of mNGS is the identification of rare and fastidious microorganisms. For example, mNGS was successfully used to diagnose severe pneumonia caused by *Chlamydophila psittaci* from DNA extracted from blood or alveolar fluid washes of patients (Chen et al., 2020). It also allows the identification of co-infections and give the opportunity to help understanding the existing interactions between different pathogens. Shotgun metagenomic techniques have been used in the diagnosis of pulmonary TB (Votintseva et al., 2017), providing information on the antibiotics susceptibility of a large proportion of the MTB strains tested. Interestingly, shotgun metagenomics, through its large and deep sequence analysis, can also reveal bacterial resistome (Mac Aogáin et al., 2020) using databases. Indeed, the implementation of genome databases, associated with their multidrug-resistance pattern, allows to quickly identifying unexpected resistance elements in the bacterial sequence of interest. Up to 47 freely available bioinformatics resources are currently proposed to the scientific community and are consistently fed through online repositories of genomic sequences and phenotypic information (e.g., minimum inhibitory concentration or disk diffusion assay). These databases allow a “sequence-based monitoring,” for example to help tracking local bacteria resistances (Hendriksen et al., 2019). They include notably: the CARD (Comprehensive Antimicrobial Resistance Database) (Jia et al., 2017), NDARO (NCBI National Database of Antibiotic Resistant Organisms), MEGARes^3^or ARDB (Antibiotic Resistance Genes Database) bioinformatics tools.

Placing NGS as a first-intention test is still under debate as results are usually obtained within 2–3 days, the technique is costly and it requires dedicated and trained people to apply it. One would suggest applying it in second intention, if patients do not respond to assigned treatment. However, clinicians have to keep in mind that this technique can offer great potential, specifically to propose personalized medicine in patients with chronic infections such as cystic fibrosis or immunocompromised patients (Lecuit and Eloit, 2015).

## Conclusion

Bacterial-associated lower respiratory tract infections are the main reason for antibiotics prescription worldwide. However, mainly due to the lack of proper diagnosis, antibiotics are often inappropriately prescribed and lead to the emergence of multi-resistant bacterial strains also called “superbugs.” One way to avoid aberrant antibiotics prescription is to shorten diagnosis process and to achieve personalized, evidence-based medicine.

In this review, we have attempted to highlight the challenges to propose accurate minimally invasive and fast-checking diagnostic methods to identify pathogens in the LRT. We have shown that newly developed diagnostics, often based on connected technologies (artificial intelligence, automated sequencing, electronics…), offer promising alternatives to traditional culture-based diagnostics. The availability of ever-growing databases on bacteria responsible for LRTs, associated with user-friendly online analysis tools, should also contribute to accelerate diagnostics. One such example is the free online tool called “MicrobeNet,” provided by the Center for Diseases Control (CDC), that lists genetic sequences, protein and biochemical profiles (enzymatic pathways and metabolism) from rare disease-causing microbes identified worldwide, in hospital and public health laboratories. Significant efforts remain to be done to propose other biomarkers for pneumonia detection and eventually strain identification.

## Author Contributions

HR, AJ, MC, AC, and ER co-wrote the manuscript. All authors contributed to the article and approved the submitted version.

## Conflict of Interest

The authors declare that the research was conducted in the absence of any commercial or financial relationships that could be construed as a potential conflict of interest.

## Footnotes

1 https://immuview.com/products/immuview-s-pneumoniae-and-legionella/

2 https://www.biomerieux.fr/diagnostic-clinique/biofire-filmarray-pneumonia-panel-plus

3 https://megares.meglab.org
